# *Lactobacillus reuteri* I5007 Modulates Intestinal Host Defense Peptide Expression in the Model of IPEC-J2 Cells and Neonatal Piglets

**DOI:** 10.3390/nu9060559

**Published:** 2017-05-31

**Authors:** Hongbin Liu, Chengli Hou, Gang Wang, Hongmin Jia, Haitao Yu, Xiangfang Zeng, Philip A. Thacker, Guolong Zhang, Shiyan Qiao

**Affiliations:** 1 State Key Laboratory of Animal Nutrition, China Agricultural University, Beijing 100193, China; binhongliu@126.com (H.L.); houchengli@163.com (C.H.); crazygang@126.com (G.W.); jiahongmin@126.com (H.J.); 15600660793@163.com (H.Y.); ziyangzxf@163.com (X.Z.); 2 Institute of Food Science and Technology CAAS, Chinese Academy of Agricultural Sciences, Beijing 100193, China; 3 Department of Animal and Poultry Science, University of Saskatchewan, Saskatoon, SK S7N 5C5, Canada; phil.thacker@usask.ca; 4 Department of Animal Science, Oklahoma State University, Stillwater, OK 74074, USA; glenn.zhang@okstate.edu

**Keywords:** *Lactobacillus reuteri*, intestinal epithelial cells, neonatal piglets, host defense peptide, gut microbiota

## Abstract

Modulation of the synthesis of endogenous host defense peptides (HDPs) by probiotics represents a novel antimicrobial approach for disease control and prevention, particularly against antibiotic-resistant infections in human and animals. However, the extent of HDP modulation by probiotics is species dependent and strain specific. In the present study, The porcine small intestinal epithelial cell line (IPEC-J2) cells and neonatal piglets were used as in-vitro and in-vivo models to test whether *Lactobacillus reuteri* I5007 could modulate intestinal HDP expression. Gene expressions of HDPs, toll-like receptors, and fatty acid receptors were determined, as well as colonic short chain fatty acid concentrations and microbiota. Exposure to 10^8^ colony forming units (CFU)/mL of *L. reuteri* I5007 for 6 h significantly increased the expression of porcine β-Defensin2 (PBD2), pBD3, pBD114, pBD129, and protegrins (PG) 1-5 in IPEC-J2 cells. Similarly, *L. reuteri* I5007 administration significantly increased the expression of jejunal pBD2 as well as colonic pBD2, pBD3, pBD114, and pBD129 in neonatal piglets (*p* < 0.05). This was probably associated with the increase in colonic butyric acid concentration and up-regulating expression of Peroxisome Proliferator-Activated Receptor-γ (PPAR-γ) and G Protein-Coupled Receptor 41 (GPR41) (*p* < 0.05), but not with stimulation of Pattern-Recognition Receptors. Additionally, supplementation with *L. reuteri* I5007 in the piglets did not affect the colonic microbiota structure. Our findings suggested that *L. reuteri* I5007 could modulate intestinal HDP expression and improve the gut health of neonatal piglets, probably through the increase in colonic butyric acid concentration and the up-regulation of the downstream molecules of butyric acid, PPAR-γ and GPR41, but not through modifying gut microbiota structure.

## 1. Introduction

On a global basis, it is estimated that 5.9 million children under the age of five years died in 2015, most of which are caused by infectious diseases associated with bacteria that are resistant to antibiotics [1]. As key components of the innate immune system, host defense peptides (HDPs) play critical roles in fighting against infections for their ability of possessing antimicrobials and a low propensity for the development of bacterial resistance in younger children with immature neonatal immune systems [2,3]. HDPs have been commonly studied for their antimicrobial properties and have been shown to kill bacteria, viruses, fungi, protozoa, and even cancer cells [3]. Due to their potential therapeutic activities, HDPs are attractive candidates as alternatives for antibiotics [4]. Swine and humans share high similarity in physiologic and anatomic characteristics, which makes the former the ideal model for human health and disease [5,6]. In vertebrate animals, HDPs are generally grouped into two major families; defensins and cathelicidins [7,8].

As an important first line of defense, HDPs are produced mainly by intestinal epithelial cells and phagocytes in the gastrointestinal tract. In addition to infection or inflammation, HDPs can also be induced by dietary compounds, including saccharides, essential amino acids, butyrate, vitamin D3, and zinc [4,9,10,11,12]. Moreover, probiotic lactobacilli could stimulate HDP expression in human cells and piglets without provoking inflammatory responses like pathogenic strains [13,14]. However, different lactobacilli strains show a varying magnitude of HDP-inducing activity [13].

*Lactobacillus reuteri* is considered to be an indigenous species in the gastrointestinal tract of humans and animals [15]. Numerous studies have demonstrated that *L. reuteri* has excellent probiotic properties and has been widely used as a probiotic in humans and animals [16]. *L. reuteri* I5007, initially known as *L. fermentum* I5007, was isolated from the colonic mucosa of healthy weaning piglets [17]. Compelling evidence shows that *L. reuteri* I5007 has several important probiotic properties including: (1) resistance to gastric acid and bile [18]; (2) strong adhesion [17,19]; (3) competitive exclusion against pathogens [19]; (4) alleviation of weaning stress in piglets [20]; (5) improvement of piglet performance [21,22]; (6) and positive regulation of redox status and immune function in piglets [23,24]. Notably, oral administration of *L. reuteri* I5007 increased the concentration of butyrate and branched chain fatty acids in the colonic digesta of suckling piglets [22,24]. It has been shown that butyrate, produced by butyrate-producing bacterial strains, has strong capacity to induce HDP expression in vitro. However, whether *L. reuteri* I5007 could modulate intestinal HDP expression through modifying gut microbiota and its metabolite butyrate in neonatal piglets is still unknown.

The aim of the current study was to investigate the effects of *L. reuteri* I5007 on the gut microbiota and HDP expression. We initially studied the in vitro effect of *L. reuteri* I5007 by inducing HDP expression in a porcine intestinal epithelial cell line. We subsequently determined the effects of *L. reuteri* I5007 supplementation on the colonic bacterial community and HDP expression in formula-fed neonatal piglets.

## 2. Materials and Methods

### 2.1. Ethics Statement

The procedures used in this experiment were approved by the China Agricultural University Institutional Animal Care and Use Committee (CAU20144-2, Beijing, China).

### 2.2. Bacterial Strain, Growth and Storage Conditions

*L. reuteri* I5007 was grown in De Man Rogosa Sharpe media under anaerobic conditions at 37 °C for 20 h. For cell culture assays, after incubation, bacterial cells were obtained by centrifugation (8000× *g* for 10 min at 4 °C). Then the bacterial cells were washed with phosphate-buffered saline (PBS, a balanced salt solution used for a variety of cell culture applications), reconstituted in DMEM/F12 (Dulbecco’s Modified Eagle Medium/Nutrient Mixture F-12, 1:1 mixture of DMEM and Ham’s F-12) medium supplemented with 10% fetal bovine serum (FBS) and adjusted to the required cell concentration. After centrifugation, the culture supernatant of *L. reuteri* I5007 was passed through a 0.2-μm-pore-size filter (Corning Inc., Corning, NY, USA), and it was preserved for subsequent treatment with a 10% (*v*/*v*) concentration. For heat killed bacteria, heat inactivation was carried out in a water bath at 65 °C for 1 h. The bacterial cells were centrifugated, and the pellet was washed with PBS and adjusted to a density of 1 × 10^8^ colony forming units (CFU)/mL with DMEM/F12 medium supplemented with 10% FBS. The freeze-dried powder, containing 5 × 10^10^ CFU/g, was produced according to Liu et al. [22].

### 2.3. Cell Culture and Treatment

The porcine small intestinal epithelial cell line (IPEC-J2) was kindly provided by Dr. Wu at Texas A & M University (College Station, TX, USA). IPEC-J2 cells were cultured in DMEM/F12 medium supplemented with 10% FBS at 37 °C in a 5% CO_2_ and 95% air atmosphere with 90% humidity.

For stimulation experiments, undifferentiated cells were seeded at a density of 1 × 10^6^ cells per well in 6-well plates (Costar, Corning Inc., Corning, NY, USA). After overnight growth (cells were grown to ~80% confluence in the culture wells), the cells were treated in duplicate with *L. reuteri* I5007. To prevent any influence of antibiotics on the immune response, the medium did not contain antibiotics. The FBS showed no effect on expression.

For dose-dependent *L. reuteri* I5007 stimulation experiments, IPEC-J2 cells were incubated with a control or 10^5^, 10^6^, 10^7^, 10^8^, or 10^9^ CFU/mL *L. reuteri* I5007 for 6 h. For time-dependent *L. reuteri* I5007 stimulation experiments, IPEC-J2 cells were incubated with 10^8^ CFU/mL *L. reuteri* I5007 for 3, 6, or 12 h.

IPEC-J2 cells were also treated for 6 h with 10^8^ CFU/mL *L. reuteri* I5007 exposed to different processing conditions. The processing conditions included a solvent control without *L. reuteri* I5007 (Control, DMEM/F12 medium supplemented with 10% FBS), 10^8^ CFU/mL live *L. reuteri* I5007 (Live I5007), 10^8^ CFU/mL heat-killed *L. reuteri* I5007 (Dead I5007, incubated in a water bath at 65 °C for 1 h), adhered *L. reuteri* I5007 (Adhered I5007, treated with 10^8^ CFU/mL *L. reuteri* I5007 for 1 h, rinsed three times in PBS with fresh medium added, followed by continued incubation for 5 h), and 200 μL of *L. reuteri* I5007-free culture supernatant of *L. reuteri* I5007 (Supernatant, diluted 1:10 in basal medium). In addition, a Transwell Insert System (Costar, Corning Inc., Corning, NY, USA) was used to avoid direct contact between the IPEC-J2 cells and *L. reuteri* I5007 (Separate I5007). Herein, *L. reuteri* I5007 cells in an upper chamber and IPEC-J2 cells in a lower chamber were separated by a 0.2-μm-pore-size filter membrane support (Corning Inc., Corning, NY, USA), thereby minimizing any direct contact between the *L. reuteri* I5007 cells and IPEC-J2.

### 2.4. Animals and Treatments

The in vivo experiment was conducted in the Metabolism Laboratory of the Ministry of Agriculture Feed Industry Centre (Beijing, China). Twenty-two, full-term, crossbred (Duroc × Large White × Landrace) male piglets, obtained from six litters, were used in this study. The piglets were delivered vaginally and allowed colostrum for 48 h after birth. The piglets were individually housed in stainless steel cages (1.4 m × 0.45 m × 0.6 m) in a temperature (32 ± 1 °C) and relative humidity (65–70%) controlled room programmed to deliver a light:dark cycle of 16:8 h.

On the third day after parturition, the piglets were trained to suckle from bottles filled with milk replacer (Jiaduonai H001, DaChan Tianyao, Tianjin, China, Table 1), which was dissolved in warm previously boiled water (45 °C, *w*/*v* 1:9). The fresh liquid milk replacer was fed to piglets individually from a feeder five times daily (6:00, 10:00, 14:00, 18:00, and 22:00 h) for 20 days. After feeding, the remaining milk was measured and the feeders were cleaned before adding new fresh milk replacer. The formula did not contain any antibiotics or other medicine.

On day 4, the neonatal piglets were allocated to one of two treatments balanced for litter of origin and body weight (initial body weight of 1.81 ± 0.31 kg) with 11 piglets assigned to each treatment (*n* = 11). The treatments were comprised of a control treatment (the piglets were given a placebo of 4 mL of 0.1% peptone) and a *L. reuteri* I5007 treatment, which involved oral administration of 1.0 × 10^10^ CFU *L. reuteri* I5007 dissolved in 4 mL of 0.1% peptone water daily for 20 days.

The health status for each piglet was recorded, and the occurrence of diarrhea was assessed two times a day (monitoring time: 10 a.m. and 4 p.m.) according to the method of Marquardt et al. [25] and Ou et al. [26]. Scores were 0 = normal, solid feces; 1 = slight diarrhea, soft and loose feces; 2 = moderate diarrhea, semi-liquid feces; or 3 = severe diarrhea, liquid and unformed feces. The occurrence of diarrhea was defined as maintaining a score of two or three for one day. The incidence of diarrhea (%) was calculated as ((number of piglets with diarrhea × number of days of diarrhea)/(total number of experiment piglets × number of days of the whole experiment)) × 100%.

On days 4, 14, and 24, the piglets were weighed. On day 24, all piglets were euthanized with Zoletil 50^®^ (Virbac, Carros, France), and all the intestinal tissues from the jejunum, ileum, and proximal distal colon were collected, frozen in liquid nitrogen, and then stored at –80 °C until total RNA was extracted. The colonic digesta were gently squeezed into sterile Eppendorf tubes, frozen in liquid nitrogen, and subsequently stored at −80 °C until processing.

### 2.5. Analysis of Porcine Gene Expression by Real Time PCR

The cells and tissues (about 0.04 mg) were lysed directly in TRIzol (Invitrogen, Carlsbad, CA, USA). Total RNA was extracted according to the manufacturer’s instructions. RNA concentrations were measured using a NanoDrop Spectrophotometer (P330, Implen, Germany). The purity was determined by the ratio of A260:A280 and A260:A230 by NanoDrop, and then the quality was checked with 1% Agarose Gel Electrophoresis following the procedures outlined by Aranda et al. [27].

The first-strand cDNA was synthesized by reverse transcription of 1 μg of total RNA using a PrimeScript 1st Strand cDNA Synthesis Kit (Takara, Dalian, China) according to the manufacturer’s protocol and stored at –80 °C. The primers used are listed in Supplemental Table S1. Porcine β-Defensin (PBD) 1, pBD2, pBD3, pBD114, pBD129, Protegrins (PG) 1-5, Epididymis Protein 2 Splicing Variant C (PEP2C), toll-like Receptors (TLR) 2, TLR4, TLR6, TLR9, Nucleotide-Binding Oligomerization Domain (NOD) 1, Mucin 1 (MUC1), Peroxisome Proliferator Activated Receptor-γ (PPAR-γ) ,G Protein-Coupled Receptor (GPR) 41, and GPR43 were determined [4,28,29,30,31].

Real-time PCR was performed on an Applied Biosystems 7500 Real-Time PCR System (Applied Biosystems, Singapore) using SYBR Green PCR Master Mix (Takara, Dalian, China). All reactions were run in triplicate. Relative gene expression was calculated according to the ΔΔC_t_ method ((C_t_ gene of interest − C_t_ internal control) treatment − (C_t_ gene of interest − C_t_ internal control) control) using porcine β-actin as the reference gene.

### 2.6. Colonic Short Chain Fatty Acid Concentrations

The concentrations of SCFA (short-chain fatty acid) were determined with a Dionex ICS-3000 Ion Chromatography System (Dionex Corporation, Sunnyvale, CA, USA) following the procedures of Qiu and Jin [32] with modification. Samples of colonic digesta (0.5 g) were weighed, diluted in a ratio of 1:5 with ultrapure water, homogenated with 8 mL ultrapure water, and then centrifuged at 10,000× *g* for 20 min at 4 °C. The supernatant was kept in a 2 mL screw-capped vial. The concentrations of formic, acetic, propionic, butyric, and lactic acid were measured with the Dionex ICS-3000 Ion Chromatography System (Dionex, Sunnyvale, CA, USA).

### 2.7. Fecal Microbiota Analysis

Microbial genomic DNA was extracted and purified from colon digesta samples using a QIAmp DNA stool mini kit (Qiagen, GmbH Hilden, Germany) modified to contain a bead-beating step. Successful DNA isolation was confirmed by agarose gel electrophoresis. PCR primers flanking the V3-V4 hyper variable region of bacterial 16S rDNA were designed. The barcoded fusion forward primer was 341F(5′-CCTAYGGGRBGCASCAG-3′), and the reverse primer was 806R(5′-GGACTACNNGGGTATCTAAT-3′). The optimized conditions for amplification were as follows: one pre-denaturation cycle at 95 °C for 5 min, 27 cycles of denaturation at 95 °C for 30 s, annealing at 55 °C for 30 s, elongation at 72 °C for 45 s, and a final extension at 72 °C for 10 min. The resulting amplicons were gel purified, quantified, pooled, and sequenced on the Illumina HiSeq 2500 platform. Microbiota sequences were processed through QIIME 1.8 (QIIME Team). After quality filtering, the sequences were denoised using denoise_wrapper.py. The denoised sequences were clustered into operational taxonomic units (OTUs) at a 97% sequence similarity against the GreenGenes OTU database (gg_13_8_otus). The chimeric OTUs were removed using UCHIME v4.2. Representative sequences for each OTU were picked and aligned using QIIME 1.8. Taxon-dependent analysis was conducted using the Ribosomal Database Project (RDP) classifier. The OTUs were counted for each sample to express the richness of bacterial species with an identity cutoff of 97%. Alpha and beta diversity calculations and taxonomic community assessments were performed using QIIME 1.8 scripts.

### 2.8. Statistical Analysis

Statistical analyses were performed using SPSS 17.0 Software (SPSS Inc., Chicago, IL, USA). All pairwise comparisons for the in vivo and in vitro data were examined using an unpaired Student’s two-tailed *t*-test. Chi square was used to test differences in diarrhea incidence between the two groups. The level of significance was set at *p* < 0.05. The results were expressed as mean ± standard error of the mean (SEM). Principle component analysis (PCA) plots were used to visualize differences in bacterial community composition among samples. The PCA plots were produced based on a euclidean metric. Linear discriminant analysis effect size (LEfSe) analysis was used to identify the OTUs or taxa, which were responsible for the differences between the groups. An effect size threshold of two was used for the biomarkers discussed in this study. The metastats program from R-script was used to identify statistically different phylotypes among groups. Only taxa with average abundances greater than 10^−3^, *p* < 0.05 and low *Q* values (low risk of false discovery) were considered significant. 

## 3. Results

### 3.1. Effects of L. reuteri I5007 on Host Defense Peptide Expression in IPEC-J2 cells

**Figure 1:**
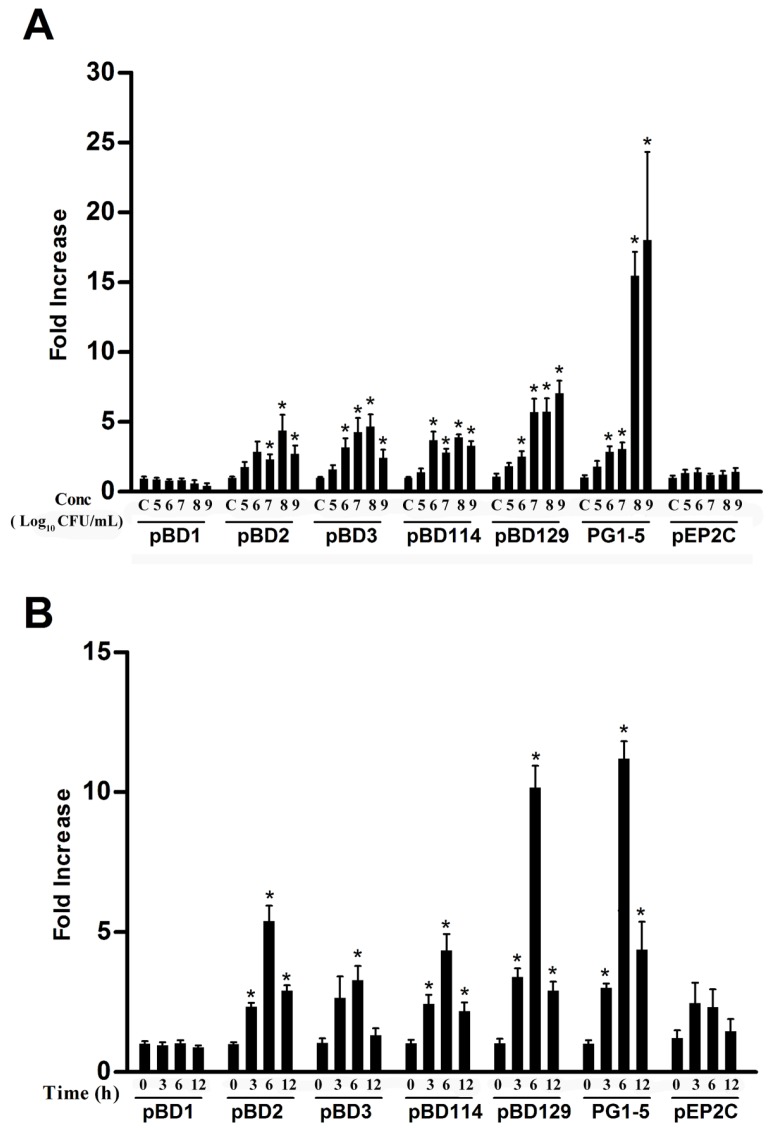
*L. reuteri* I5007-induced expression of pBD1, pBD2, pBD3, pBD114, pBD129, PG1-5, and pEP2C in porcine IPEC-J2 cells. Cells were incubated in duplicate with the indicated concentrations of (**A**) *L. reuteri* I5007 for 6 h or (**B**) 10^8^ CFU/mL for 3, 6, or 12 h. Gene expression was analyzed by real-time PCR. The relative fold changes over the unstimulated control were calculated with the ΔΔC_t_ method using the β-actin gene for normalization. Data are mean ± standard error obtained in three independent experiments. “C” in X-axis, control group. * *p* < 0.05 by unpaired Student’s *t*-test. pBD, porcine β-Defensin; PG, protegrins; pEP2C, Epididymis Protein 2 Splicing Variant C; IPEC-J2, The porcine small intestinal epithelial cell line; CFU, colony forming units.

The mRNA expressions of porcine HDP, including pBD1, pBD2, pBD3, pBD114, pBD129, PG1-5, and pEP2C, were determined in IPEC-J2 cells to study the effects of *L. reuteri* I5007 on the modulation of HDP. First the dose-dependence of HDP gene expression following treatment of IPEC-J2 cells with *L. reuteri* I5007 was examined. Our results indicate that a 6 h treatment with *L. reuteri* I5007 markedly increased the mRNA expression of pBD2, pBD3, and pBD114 in a dose-dependent manner in IPEC-J2 cells, peaking at 10^8^ CFU/mL (Figure 1A). The mRNA expression levels of pBD129 and PG1-5 were also dose-dependently induced by *L. reuteri* I5007 in IPEC-J2 cells, with the maximal response occurring at 10^9^ CFU/mL (Figure 1A). However, the magnitude of induction varied obviously among the five genes, with PG1-5 showing an approximately 17-fold increase, whereas pBD3, pEP2C, and PG1-5 showed only 5-, 5-, and 7-fold induction at the peak response, respectively (Figure 1A).

An obvious time-dependent induction of pBD2, pBD3, pBD114, pBD129, and PG1-5 was also observed in IPEC-J2 cells after 10^8^ CFU/mL *L. reuteri* I5007 treatment (Figure 1B). The maximum HDP mRNA was expressed after 6 h of incubation and decreased at 12 h of incubation. It is noteworthy that pBD1 and pEP2C were largely unaltered in the IPEC-J2 cells following *L. reuteri* I5007 treatment.

### 3.2. Effects of Different Processing Conditions on L. reuteri I5007 Induced Host Defense Peptide Expression in IPEC-J2 Cells

**Figure 2:**
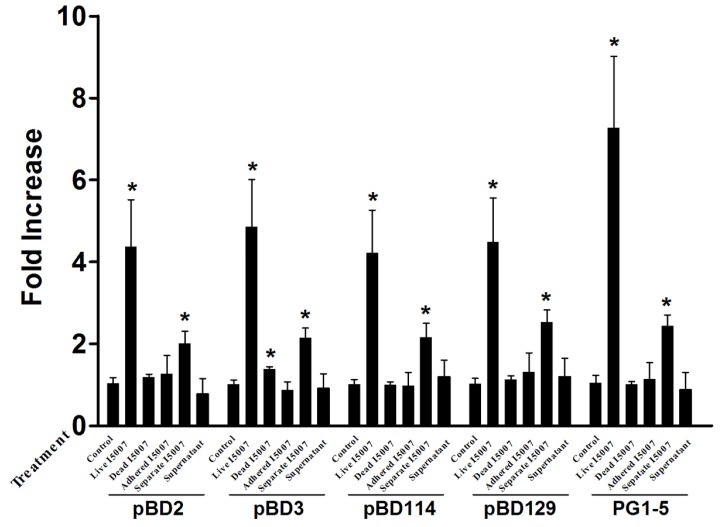
Regulation of pBD2, pBD3, pBD114, pBD129, and PG1-5 expression by *L. reuteri* I5007 subjected to different processing conditions. Porcine IPEC-J2 cells were incubated with 10^8^ CFU/mL *L. reuteri* I5007 (Live I5007), heat-killed *L. reuteri* I5007 (Dead I5007), adhered *L. reuteri* I5007 (Adhered I5007), *L. reuteri* I5007 without direct contact with IPEC-J2 (Separate I5007), culture supernatant of *L. reuteri* I5007 (Supernatant), and the solvent control (Control). Gene expression was analyzed by real-time PCR. The relative fold changes over the unstimulated control were calculated with the ΔΔC_t_ method using the β-actin gene for normalization. Data are mean ± standard error obtained in three independent experiments. * *p* < 0.05 by unpaired Student’s *t*-test.

To further examine whether *L. reuteri* I5007 induced HDP mRNA expression is altered by different processing conditions, IPEC-J2 cells were treated for 6 h with *L. reuteri* I5007 produced under different processing conditions (Figure 2). We studied whether a heat-killed strain had the ability to stimulate the expression of HDP. The results indicated that heat-killed *L. reuteri* I5007 was much less effective than the live strain and only stimulated pBD3 expression.

According to a previous study conducted in our lab, *L. reuteri* I5007 has strong adhesion ability to monolayer cells when co-cultured with cells for 1 h [14]. IPEC-J2 cells were treated with *L. reuteri* I5007 for 1 h, non-adherent bacteria were washed away, and incubation continued for a further 5 h. The results revealed that the adherent bacteria were insufficient for *L. reuteri* I5007-induced HDP expression.

To further examine whether cell-to-cell contact is required for *L. reuteri* I5007 to stimulate HDP expression in IPEC-J2 cells, a Transwell Insert System, in which the bacteria and host cells are partitioned by a 0.22-μm membrane, was used to prevent all direct cell-to-cell contact between *L. reuteri* I5007 and IPEC-J2 cells. Under these conditions, *L. reuteri* I5007 enhanced the mRNA expression of pBD2, pBD3, pBD114, pBD129, and PG1-5 (Figure 2). These findings indicate that direct cell-to-cell contact is not required for *L. reuteri* I507 to stimulate HDP expression, and that some metabolite produced by *L. reuteri* I5007 may be able to cross the membrane and induce HDP expression.

To further elucidate whether the culture supernatant was involved in *L. reuteri* I5007 induced HDP expression, a culture supernatant of *L. reuteri* I5007 was also added to IPEC-J2 cells. Compared with the control group, cells treated with *L. reuteri* I5007 culture supernatant demonstrated no significant change in the mRNA expression of HDP. These findings indicate that there are no compounds in the culture supernatant without *L. reuteri* I5007 that stimulate HDP production in IPEC-J2 cells.

### 3.3. Effects of L. reuteri I5007 on Neonatal Piglet Performance

In this study, pigs in the two treatments (*L. reuteri* I5007 group and control group) started at the same age (day 4) and had similar body weights (*p* = 0.99) (Table 2). At the end of the experimental period (day 24), the piglets treated with *L. reuteri* I5007 had 15.07% higher (*p* < 0.05) average daily gain than control piglets. Moreover, the diarrhea incidence and diarrhea scores were lower in piglets administrated with *L. reuteri* I5007 compared with the control, although not significant (3.64 vs. 5.91%, 0.12 vs. 0.20). 

### 3.4. Effects of L. reuteri I5007 on Host Defense Peptide Expression in Neonatal Piglets

**Figure 3:**
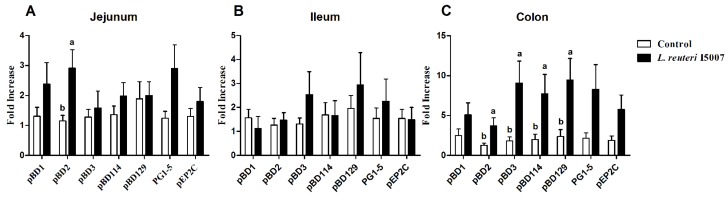
Regulation of pBD1, pBD2, pBD3, pBD114, pBD129, PG1-5, and pEP2C expression by *L. reuteri* I5007 in the (**A**) jejunum, (**B**) ileum, and (**C**) colon of neonatal piglets. Twenty-two male neonatal piglets were orally administrated with 0.1% peptone solution (control) or 1 × 10^10^ CFU of *L. reuteri* I5007 daily for 20 days. Gene expression was analyzed by real-time PCR. The relative fold changes over the control were calculated with the ΔΔC_t_ method using the β-actin gene for normalization. White bars or black bars represent control or *L. reuteri* I5007 treatments, respectively. Values are presented as mean ± standard error of the mean, *n* = 11 piglets per treatment. Bars with different letters differ, *p* < 0.05 by unpaired Student’s *t*-test.

Twenty-two male neonatal piglets were orally administrated with 0.1% peptone solution or 1 × 10^10^ CFU of *L. reuteri* I5007 daily for 20 days. The levels of mRNA expression of pBD1, pBD2, pBD3, pBD114, pBD129, PG1-5, and pEP2C in the jejunum, ileum, and colon were measured and are presented in Figure 3. Compared with the control group, no significant difference was observed in the ileal HDP expression in the *L. reuteri* I5007 group. Only pBD2 expression in the *L. reuteri* I5007 group was observed to be significantly higher than that of the control group in the jejunum. However, pBD2, pBD3, pBD114, and pBD129 mRNA expression were significantly up-regulated in the colon of piglets administrated with *L. reuteri* I5007 compared with the control piglets.

### 3.5. Effects of L. reuteri I5007 on Short Chain Fatty Acid Concentrations in Colonic Digesta

The concentrations of SCFA in colonic digesta are presented in Table 3. The concentration of butyric acid was higher in piglets treated with *L. reuteri* I5007 compared with the control group (*p* < 0.05). The concentration of formic acid tended to be higher in *L. reuteri* I5007 treated piglets (*p* = 0.08), while the acetic, propionic, and lactic acid concentrations did not differ between the two treatments.

### 3.6. Effects of L. reuteri I5007 on PPAR-γ, GPR41 and GPR43 in Colonic Tissue

**Figure 4:**
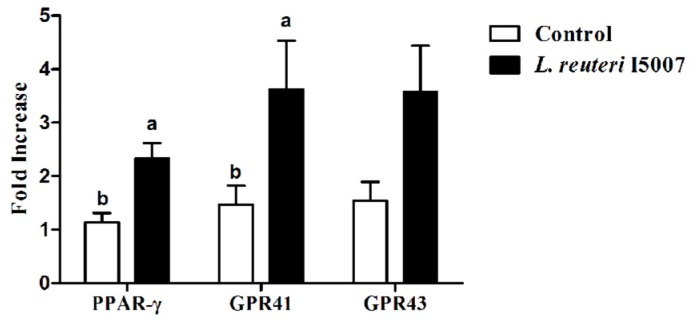
Effects of *L. reuteri* I5007 on PPAR-γ, GPR41, and GPR43 expression in the colonic tissue of neonatal piglets. Gene expression was analyzed by real-time PCR. The relative fold changes over the control were calculated with the ΔΔC_t_ method using the β-actin gene for normalization. Data are mean ± standard error (*n* = 11). Bars with different letters differ, *p* < 0.05 by unpaired Student’s *t*-test.

The relative transcription levels of PPAR-γ, GPR41, and GPR43 in the colonic tissue were analyzed using real-time PCR (Figure 4). The relative abundances of mRNA for PPAR-γ and GPR41 were significantly increased in the colonic tissue of piglets treated with *L. reuteri* I5007 compared with the control treatment (*p* < 0.05).

### 3.7. Effects of L. reuteri I5007 on Bacterial Community Structure in Colonic Digesta

**Figure 5:**
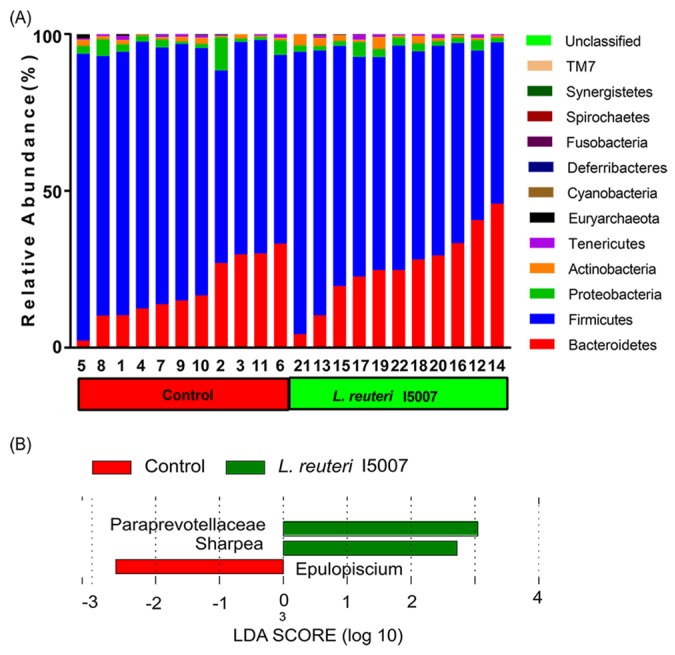
Effects of *L. reuteri* I5007 on bacterial community structure in colonic digesta. (**A**) Relative abundance levels of the bacterial phyla present in control group and *L. reuteri* I5007 group; (**B**) Histogram of the LDA scores computed for taxa differentially abundant between control and *L. reuteri* I5007 treated piglets (*n* = 11).

A total of 810,187 high quality sequences were obtained from all fecal samples, with an average of 36,826 sequences per sample. These sequences were assigned to 680 operational taxonomic units (OTUs). The Shannon diversity indices reached stable values, suggesting that the present study captured the dominant phylotypes. The fecal samples of all of the pigs were dominated by four phyla; *Bacteroidetes*, *Firmicutes*, *Spirochaetes*, and *Proteobacteria*, with *Firmicutes* and *Bacteroidetes* accounting for >95% of the bacteria (Figure 5A). The abundance of *Firmicutes* (76.69 ± 3.16 vs. 69.41 ± 3.46; *p* = 0.13, *Q* = 0.87) and *Bacteroidetes* (18.26 ± 3.04 vs. 25.81 ± 3.63; *p* = 0.13, *Q* = 0.87) was not significantly different between groups (Supplemental Table S2). An analysis of colonic microbiota composition by principal component analysis revealed that *L. reuteri* I5007 administration did not affect the overall composition of the fecal microbiota (Supplemental Figure S1). No significant differences were observed in relative abundance of bacterial taxa or operational taxonomic units between placebo-treated and *L. reuteri*–treated piglets except that three bacterial taxa were identified by LEfSe analysis (linear discriminant analysis, LDA score >2) (Figure 5B). These significant differences were further confirmed by metastats analysis, in which genera *Sharpea* were significantly increased in *L. reuteri* I5007 group (*p* = 0.03 and 0.02, respectively) (Supplemental Table S2). To determine whether the difference in colonic butyrate concentration was caused by the alteration of genera *Sharpea*, the correlation between the butyrate level and *Sharpea* was analyzed. However, no significant correlation was found between the butyrate level and genera *Sharpea* (*r* = 0.05, *p* = 0.82, Spearman). 

### 3.8. Effects of L. reuteri I5007 on Pattern-Recognition Receptor (PRRs) Expression in Intestinal Tissue 

An analysis of PRRs by real-time PCR revealed that the expressions of TLR2, TLR4, TLR6, TLR9, and NOD1 were not changed with *L. reuteri* I5007 treatment in the jejunum, ileum, or colon (*p* > 0.05). The relative abundance of mRNA for MUC1 was significantly increased in the colon with *L. reuteri* I5007 treatment compared with the control treatment (*p* < 0.05), while no significant differences were observed in the jejunum and ileum (Supplemental Figure S2).

## 4. Discussion

In the present study, we observed that the probiotic *L. reuteri* I5007 induced the expression of HDP in a porcine small intestinal epithelial cell line (IPEC-J2). In addition, we observed that oral administration of *L. reuteri* I5007 stimulated colonic HDP expression in neonatal piglets. Finally, we found that *L. reuteri* I5007 increased concentrations of butyric acid in neonatal piglets but did not affect the colonic bacterial community structure.

Probiotics have been shown to induce β-Defensin (hBD-2) in human cells, and different probiotic strains show different HDP-inducing activity [13]. In pigs, *L. salivarius* can induce pBD2 production in the digestive tract [14]. *L. reuteri* is one of dominant species in the gastrointestinal tracts of humans and animals and is currently used as a probiotic in pigs [16]. Oral administration of *L. reuteri* modulates ileum microbial composition, intestinal development, and immune status in pigs [22,24]. However, there are no reports of the effects of *L. reuteri* on the stimulation of HDP gene expression in IPEC-J2 cells and pigs.

Previous studies have indicated that pBD1, pBD2, pBD3, pBD114, pBD129, PG1-5, and pEP2C are expressed in IPEC-J2 cells [4]. In this study, we found that *L. reuteri* I5007 administration increased pBD2, pBD3, pBD114, pBD129, and PG1-5 gene expression in these same cells. The time-dependent experiments showed a similar pattern, as previously described by Wehkamp et al. [33] and Schlee et al. [13], with the maximum level of HDP being induced after 6 h of incubation. The dose-dependent experiment showed that *L. reuteri* I5007 induced HDP production when the concentration of *L. reuteri* I5007 reached 10^6^ CFU/mL.

Previously, Schlee et al. [13] used heat-killed bacteria, but we decided to research the effects of live strains. Since *L. reuteri* I5007 is used as a probiotic, the results for live strains may be helpful for future in vivo studies. Wehkamp et al. [33] also found a living form of *E. coli* Nissle 1917, which showed a strong induction of hBD-2 after incubation with Caco-2 cells for 4.5 h. A previous study demonstrated that heat-killed bacteria induced hBD-2 [13], but we found that only pBD3 expression was significantly increased with heat killed *L. reuteri* in the present study. Compared with the live strain, the capacity of the heat-killed *L. reuteri* I5007 to induce HDP was visibly diminished.

The suspension without bacteria did not induce HDP, which is consistent with the findings described by Wehkamp et al. [33] for *E. coli* Nissle 1917. In order to determine whether *L. reuteri* I5007 induced HDP by cell-to-cell contact, a Transwell Insert System was used. Our results indicate that *L. reuteri* I5007 without contact with IPEC-J2 cells also induced HDP, which suggests that a metabolite produced by *L. reuteri* I507 may be playing a role.

Neonatal piglets have an immature immune system, are susceptible to infections, and often suffer from diarrhea and growth retardation if infected [4]. Our previous studies showed that the optimum dosage (about 10^10^ CFU/day of *L. reuteri* I5007) could improve performance and reduce diarrhea incidence in neonatal piglets [22]. In this study, we obtained a similar result. We also found that the administration of *L. reuteri* I5007 induced pBD2 in the jejunum and pBD2, pBD3, pBD114, and pBD129 in the colon. Similar results have been obtained for *L. salivarius* induced expression of pBD2 in the pig jejunum [14]. The spatial heterogeneity patterns of this induction effect were probably due to the production of butyric acid, mainly triggered in the hindgut after *L. reuteri* I5007 administration [34]. It has been reported that most of the porcine HDP (e.g., pBD1, pBD2, pBD114, pBD129, PG1-5, pEP2C) show various activity against Gram-negative and Gram-positive bacteria, including *Salmonella typhimurium*, *Escherichia coli*, and *Clostridium perfringen*, the blooms of which are involved in the occurrence of diarrhea [35,36]. In addition to its antimicrobial properties, pBD3 could also regulate the expression of IL-8 and intestinal tight junction protein and exhibits a strong immunoregulatory ability [37]. In addition, β-defensins (hBD2) have been observed to have the capacity to recruit leukocytes. These actions can directly modify the inflammatory response [38]. The induction of the expression of HDP genes allows the immature intestinal epithelial surfaces of neonatal piglets to cope with these continuously complex microbial challenges [38]. Furthermore, previous studies showed that the supplementation of synthetic HDP could improve nutrient digestibility, intestinal morphology, and growth performance in weanling pigs [39] and broiler chickens [40]. These results suggest that the induction of HDP gene expression by *L. reuteri* I5007 may be responsible for the body weight increase and decrease in diarrhea incidence, which was mostly caused by the bloom of pathogens that can be eliminated by the HDPs [35,41,42].

Short chain fatty acids are the major metabolites of microbial digestion in the colon and have been considered as contributing an important role in normal colonic morphology and function [34]. Recent published data have proven that HDP could be induced by SCFA, especially by butyrate [4]. Our previous work and that of others indicated that administration of probiotics increased butyrate levels in the colonic digesta and fecal samples [22,24]. In this experiment, after the administration of *L. reuteri* I5007, the quantity of butyric acid in the colonic digesta was increased, which is consistent with the finding of Liu et al. [22] and may be the mechanism through which *L. reuteri* I5007 induces the expression of HDP.

Exposure to SCFA, such as butyrate, triggers profound changes in epithelial gene expression in vitro [43,44], which are mediated at least in part through the SCFA sensor PPAR-γ [45]. GPR41 (Free Fatty Acid Receptor 3, FFA3) and GPR43 (FFA2) are related G Protein-Coupled Receptors that are activated by short chain carboxylic acids [46,47]. In order to further confirm the changes of SCFA, we determined the changes of short-chain fatty acid receptors in the colon. PPAR-γ and GPR41 were observed to be higher in mRNA expression after *L. reuteri* I5007 administration compared with piglets in the control treatment.

Changes in the structure of gut microbiota alter the gut-microbial metabolism and eventually influence intestinal mucosal immunity and host metabolism [48,49]. Previous studies have reported increases of lactic acid bacteria after the intake of individual lactobacilli strains [50,51,52,53]. The administration of *Lactobacillus rhamnosus* GG increases the fecal butyrate level through expanding butyrate-producing bacterial strains [54]. To examine whether the significant difference in butyrate concentration of colonic digesta was induced by the change of microbiota structure after *L. reuteri* I5007 administration, the colonic microbial community was determined. Unexpectedly, the results of the next generation high throughput sequencing showed no significant changes in the colonic microbiota composition or stability, which was consistent with our previous finding using polymerase chain reaction (PCR)-denaturing gradient gel electrophoresis (DGGE) profiling [22]. Similar observations have been reported in a recent review [55], suggesting that probiotics do not significantly modify the gut microbiota composition of healthy subjects. Additionally, microbiomes were compared at different taxonomic levels, and no differences were detected except the specific increase of genera *Sharpea*, which is a member of *Clostridium XVII* and exhibits a close phylogenetic association with *Lactobacillus catenaformis* and *Lactobacillus vitulinus* [56]. However, no significant correlation was found between the butyrate level and genera *Sharpea*, which indicated that the increase of butyrate levels was not due to the alteration of genera *Sharpea*. It has recently been reported that the introduction of probiotics significantly changes the microbiome’s transcriptional profile but has no significant impact on the structure, leading us to speculate that *L. reuteri* I5007 may increase the butyrate concentration through modulating the microbial metabolic activities. Taken together, these results suggest that *L. reuteri* I5007 might increase butyrate levels through modulating the microbiota at the transcript level, rather than modifying the bacterial community structure in the colonic digesta [57,58]. PRRs could recognize conserved molecular motifs present on a wide range of different microbes, which have been termed Microbe-Associated Molecular Patterns (MAMPs). TLRs and NOD proteins are two classes of PRRs involved in innate immune detection [59]. Agreeing with the result of colonic bacterial community analysis, the mRNA levels for PRRs were unaffected by *L. reuteri* I5007 treatment, which indicated that *L. reuteri* I5007 induction of HDP expression was not induced through regulating the structure of the colonic bacterial community or expression of PRRs [57,58,60].

## 5. Conclusions

In conclusion, our study indicates that *L. reuteri* I5007 could regulate the expression of pBD2, pBD3, pBD114, pBD129, and PG1-5 in IPEC-J2 cells and stimulate the mRNA expression of colonic pBD2, pBD3, pBD114, and pBD129 in the neonatal piglets, which is probably mediated by increased butyric acid production and the up-regulation of the downstream molecules of butyric acid, PPAR-γ and GPR41, but not by modulation of the colonic microbial community. These findings suggest that the probiotic *L. reuteri* I5007 enhances HDP expression, which strengthens the mucosal antimicrobial barrier of neonatal piglets and is one explanation for the growth promoting effect of *L. reuteri* I5007. The identification of *L. reuteri* I5007 with favorable odors will expedite its application as a non-antibiotic, immune boosting additive for infectious disease prevention and control in pigs and other animal species; perhaps human beings as well.

## Figures and Tables

**Table 1:** Composition and nutrient levels of the experimental diets (%, as-fed basis) ^1^.

Items	Content
Crude protein	24.78
Gross energy (MJ/kg)	20.46
Lactose	35.10
Calcium	0.92
Total phosphorus	0.73
The analyzed contents of amino acids in diets	
Asparate	2.58
Threoline	1.72
Serine	1.35
Glutamate	4.52
Proline	1.54
Glycine	0.55
Alanine	1.30
Valine	1.48
Isoleucine	1.42
Leucine	2.54
Tyrosine	0.81
Phenylalanine	0.92
Histidine	0.52
Lysine	2.03
Methonine	0.65
Argine	0.76

^1^ Values are the means of a chemical analysis conducted in duplicates.

**Table 2:** Effects of *L. reuteri* I5007 on neonatal piglet performance and diarrhea incidence ^1^

Items	Control	*L. reuteri* I5007	SEM	*p* Value
Body weight at day 4 (kg)	1.81	1.81	0.07	0.99
Body weight at day 14 (kg)	2.45	2.50	0.07	0.72
Body weight at day 24 (kg)	3.72	4.00	0.10	0.16
Average daily gain (g)				
4–14 days	64	69	3.91	0.52
14–24 days	127 ^b^	151 ^a^	4.86	0.01
4–24 days	96 ^b^	110 ^a^	3.31	0.03
Average feed intake (g/day)				
4–14 days	53	58	3.43	0.08
14–24 days	140	152	5.25	0.82
4–24 days	97	105	3.24	0.18
Diarrhea score ^2^	0.20	0.12		
Diarrhea incidence ^3^ (%)	5.91	3.64		0.32

^a,b^ Means within a row with different superscripts are significantly different (*p* < 0.05). ^1^ SEM, standard error of the mean, *n* =11 for each treatment. ^2^ Diarrhea scores were 0 = normal, solid feces; 1 = slight diarrhea, soft and loose feces; 2 = definitely unformed, moderately fluid feces; or 3 = very watery and frothy diarrhea; ^3^ The occurrence of diarrhea was defined as maintaining a score of 2 or 3 for one day. The incidence of diarrhea (%) was calculated as ((number of piglets with diarrhea × number of days of diarrhea)/(total number of experiment piglets × number of days of the whole experiment)) × 100%.

**Table 3:** Effects of *L. reuteri* I5007 on short chain fatty acid concentrations (mmol/kg, wet weight) in colonic digesta obtained from neonatal piglets ^1^.

Fatty acid	Control	*L. reuteri* I5007	SEM	*p* Value
Formic acid	0.03	0.03	0.01	0.66
Acetic acid	32.49	37.74	2.03	0.20
Propionic acid	12.99	15.90	0.83	0.08
Butyric acid	7.10 ^b^	9.51 ^a^	0.58	0.04
Lactic acid	3.67	3.66	0.35	1.00

^a,b^ Means within a row with different superscripts are significantly different (*p* < 0.05). ^1^ SEM, standard error of the mean, *n* =11 for each treatment. Means within a row with different superscripts are significantly different (*p* < 0.05).

## Supplementary Materials

The following are available online at http://www.mdpi.com/2072-6643/9/6/559/s1. Figure S1: Effects of *L. reuteri* I5007 on bacterial community structure in colonic digesta, Figure S2: Effects of *L. reuteri* I5007 on TLR2, TLR4, TLR6, TLR9, NOD1, and Muc1 expression in the (A) jejunum, (B) ileum, and (C) colon of neonatal piglets, Table S1: Primer sequences used in the study, Table S2: Effect of *L. reuteri* I5007 on the relative abundance (%) of bacterial groups at the phylum, family, and genus level (above 1% abundance in at least one sample) detected in colonic digesta microbiota of piglets (*n* = 11).

## Abbreviations

HDP, host defense peptide; pBD, porcine β-defensin; PPAR-γ, peroxisome proliferator-activated receptor-γ; GPR, G protein-coupled receptor; SCFAs, short chain fatty acids; LDA, linear discriminant analysis; CFU, colony forming units; PRRs, pattern-recognition receptors; pEP2C, porcine epididymis protein 2 splicing variant C; PG, cysteine-rich protegrins; TLR, toll-like receptor; NOD, nucleotide-binding oligomerization domain; MUC, Mucin.
