# Supplementary material for: Lactobacillus reuteri I5007 Modulates Intestinal Host Defense Peptide Expression in the Model of IPEC-J2 Cells and Neonatal Piglets
Source: Nutrients. 2017 May 31;9(6):559. doi: 10.3390/nu9060559 (PMC5490538; doi:10.3390/nu9060559)
Supplement: Supplementary file 1 [file nutrients-09-00559-s001.docx]

**Supplemental Figure S1.** Effects of *L. reuteri* I5007 on bacterial community structure in colonic digesta. Communities clustered using principal coordinates analysis of the euclidean metric (n = 11).

**Supplemental Figure S2.** Effects of *L. reuteri*I5007 on TLR2, TLR4, TLR6, TLR9, NOD1 and Muc1 expression in jejunum (A), ileum (B) and colon (C) ofneonatal piglets. Gene expression was analyzed by real-time PCR. The relative fold changes over the control were calculated with the △△Ct method using the β-actin gene for normalization. Data are mean ± standard error of the mean (n = 11). Bars with different letters differ, *P* < 0.05 by unpaired Student’s t-test.

**Supplemental Table S1**. Primer sequences used in the study.

| **Supplemental Table S2**. Effect of L. reuteri I5007 on the relative abundance (%) of bacterial groups at phylum, family and genus level(above 1% abundance in at least one sample) detected in colonic digesta microbiota of piglets (n = 11). |
| --- |
